# The importance of incorporation of real-world evidence into the guidelines on the pharmacological treatment of schizophrenia

**DOI:** 10.1007/s00406-024-01938-1

**Published:** 2024-11-13

**Authors:** Istvan Bitter, Lajos Katona, Peter Falkai, Pal Czobor

**Affiliations:** 1 Department of Psychiatry and Psychotherapy, Semmelweis University, Budapest, Hungary; 2 German Center for Mental Health (DZPG), Munich, Germany; 3 Department of Psychiatry and Psychotherapy, School of Medicine, Ludwig-Maximilians-University of Munich, 13 Munich, Germany

**Keywords:** Schizophrenia, Guidelines, Real-world evidence, Real-world data, Randomized clinical trials

## Abstract

The quantity and quality of real-world data and real-world evidence in schizophrenia research are at a high level. However, these results are not included in the grading systems used to develop treatment guidelines for schizophrenia. A meta-analysis and a network meta-analysis have independently provided evidence that the results of randomized clinical trials in schizophrenia adequately translate to real-world settings. The authors propose the incorporation of a synthesis of evidence derived from analyses of randomized controlled trials and real-world data as a novel and highest level of evidence in grading instruments used to develop treatment guidelines for schizophrenia.

## Introduction

Regulatory agencies, such as the European Medicines Agency (EMA) and the U.S. Food and Drug Administration (FDA), frequently use real-world evidence (RWE) based on real-word data (RWD) to evaluate the safety and efficacy of medicines, and are seeking to better integrate these data into their decision-making processes [1, 2]. The use of RWE and RWD for regulatory decisions is more advanced “…when the course of the disease is predictable (e.g., certain rare diseases and cancers), and the effect of the drug is substantial.” [3]. The large variability in the course of schizophrenia alone is a significant limitation to the development of similar regulatory decision processes for schizophrenia.

Schizophrenia treatment guidelines provide recommendations for the treatment of patients in real-world settings based on well-defined hierarches of evidences. Most grading instruments used for the development of pharmacologic treatment guidelines for schizophrenia consider “high-quality meta-analyses, systematic reviews of RCTs, or RCTs with a very low risk of bias” to be the highest level of evidence [4]. However, none of the widely available schizophrenia treatment guidelines included in their rating system a subscale or item for RWD&RWE (for review see [5]). One of the authors (IB) recently reviewed schizophrenia guidelines from seven European countries (Finland, Germany, Norway, Slovakia, Switzerland, Ukraine, United Kingdom) with the help of a standardized tool [6]. None of them addressed the need to revise the grading systems, but we can find specific recommendations in some of the guidelines that are based solely on RWS.

Real-world studies (RWS) have been published about treatment outcomes in schizophrenia in an increasing number in last ca. 25 years. At the beginning of this century large observational clinical effectiveness studies on second generation antipsychotics were performed, such as CATIE (Clinical Antipsychotic Trials of Intervention Effectiveness), CUtLASS (Cost Utility of the Latest Antipsychotic Drugs in Schizophrenia Study), SOHO (Schizophrenia Outpatient Health Outcomes) and ZODIAC (Ziprasidone Observational Study of Cardiac Outcomes). They were called real-world studies [7]. The meaning of RWS, RWD, and RWE evolved over time, particularly in response to the computerization in healthcare. However, no universally accepted definitions currently exist. In a recent paper authors affiliated with the FDA defined it as: “Real-world data can be defined as data relating to patient health status or the delivery of health care routinely collected from a variety of sources, such as the electronic health record and administrative data.” [3]. A recent definition of RWD encompasses a range of study designs, including randomised trials with pragmatic features, non-randomised interventional studies and non-randomised non-interventional studies [8].

New insights into the relationship between evidence from randomised clinical trials (RCTs) and evidence from RWS have recently been the subject of two papers [9, 10]. The aim of this short communication is to provide a focused review of these two studies.

## Method

We conducted a search of the Pubmed database (February 2024) and subsequently repeated the search on August 4, 2024. Our search string included the keywords "real-world AND RCT AND antipsychotic," which were also utilized by Efthimiou et. al. [10] in their investigation. We explored Pubmed with additional keywords for further publications on this topic. A further search of PubMed was conducted using additional keywords in order to identify any additional publications on this topic.

## Results

Our searches consequently yielded only two relevant results: one meta-analysis [9] and one network meta-analysis (NMA) [10]. An additional publication included a meta-analysis focusing on both observational studies and RCTs, but it compared only olanzapine with a limited number of antipsychotics; the authors reported no significant differences between the results of RCTs and observational studies [11].

The meta-analytic study by Katona et al. [9] was based on 11 RWS, incorporating the aggregate data from a total of 88,148 patients’ records. The endpoint was time to all-cause treatment discontinuation (in case this was not available for RCTs, the drop out from the trial was investigated). For RCTs, the 7 prior published meta-analyses that were used as reference covered a total of 27,765 patients. Eight selected antipsychotic medications were included in the meta-analysis (one long-acting injectable (LAI) and seven oral formulations), conducting a head-to-head comparison of 25 pairs of antipsychotics. The overwhelming majority of RWS yielded statistically conclusive differences between individual antipsychotic pairs (13 out of the 17 comparisons summarising 3 or more results from RWS) and, clinically even more importantly, consistent findings across individual RWS (12 out of the 17 comparisons summarising 3 or more results from RWS). The main finding was that in most of the pair-wise comparisons there was a good congruency between the results of RWS and RCTs (in 9 out of 12 comparisons where RCT results were available).

The NMA of Efthimiou et. al [10] compared the efficacy and effectiveness of antipsychotics for relapse prevention in schizophrenia and estimated overall treatment effects using all available RCTs and RWE. The authors included 90,469 individuals from Swedish and Finnish registries and 10,091 individuals from 30 RCTs. They found no evidence of differences between effectiveness (RWD) and efficacy (RCTs) using between-drug comparisons, apart from superior effectiveness of LAI versus oral antipsychotics in RWD compared to RCTs. The authors reported that their RCT NMA exhibited heterogeneity and inconsistency. The results of their NMA are of particular importance, since they constitute an independent replication of the principal findings and conclusions of the Katona et al. study, which: “…provides empirical evidence that the RCT data adequately translate to clinical settings" [9].

## Discussion

The two studies used somewhat different methodologies, which both have their advantages and disadvantages. Efthimiou et. al applied NMA, and utilized individual patient records [10] that theoretically can provide better precision than the analysis based on the aggregate data used by Katona et al. [10]. However, the NMA approach may suffer from the problems of generalizability, since some of the studies with no individual patient records available were not included in their analyses due to the methodology. Furthermore, observational studies reflecting a broader coverage of prescribing patterns outside of Swedish and Finnish registries were not included, as pointed out by Ostuzzi & Barbui [12], who emphasized the importance of the impact of differing prescribing practices under various clinical scenarios. While the NMA adopted by Efthimiou et. al [10] for their analyses represents an innovative statistical approach, “Further research is needed to clarify whether end-users who do not have specialist statistical knowledge can assess the quality and validity of evidence produced in systematic reviews using NMA methods, even with a critical appraisal tool optimised for such studies.” [13].

The findings from the two studies converge to the same conclusion that Katona et al. [9] summarized as “*our study provides empirical evidence that the RCT data adequately translate to clinical settings*", and Efthimiou et. al [10] summarized as “*antipsychotic between-drug comparison findings for the outcome of relapse prevention might be portable from RCTs to the real world*”. In addition, it is also important that for “face to face” comparisons of antipsychotics that lack empirical data from RCTs, RWE can provide invaluable guidance for clinicians. They can also help formulate clinically relevant hypotheses for testing in subsequent RCTs, thereby filling existing knowledge gaps for clinical practice. It would be possible to include RWE into grading systems for guidelines that recommend the use of a multi-step approach of defining levels of evidence, applying criteria for their grading and the grading of recommendations e.g. [14].

## Conclusions

Overall, the results from the Katona et al.’s [9] and the Efthimiou et. al’s [10] studies underline the importance and timeliness of the synthesis of the evidence from randomized controlled and real world studies. The current level of evidence from RWS, especially when combined with results from RCTs, merits consideration for inclusion in the evidence grading instruments of guidelines alongside RCTs, case–control or cohort studies, case reports/series, and expert opinion [4]. The authors propose the inclusion of combined evidence from meta-analyses and network meta-analyses of RWS and RCTs as a new, highest level of evidence in grading instruments used for schizophrenia treatment guidelines. With the introduction of living schizophrenia treatment guidelines, the inclusion of RWE could significantly increase the value of the guidelines to practitioners with a broader range of comparative effectiveness and safety data for antipsychotics and other medications.
